# Premature Ovarian Insufficiency and Diminished Ovarian Reserve: From Diagnosis to Current Management and Treatment

**DOI:** 10.3390/jcm14217473

**Published:** 2025-10-22

**Authors:** Lara Houeis, Jacques Donnez, Marie-Madeleine Dolmans

**Affiliations:** 1 Gynecology Research Unit, Institut de Recherche Experimentale et Clinique, Université Catholique de Louvain, 1200 Brussels, Belgium; lara.houeis@uclouvain.be; 2 Society for Research into Infertility, 1150 Brussels, Belgium; jacques.donnez@gmail.com; 3 Gynecology Department, Cliniques Universitaires Saint-Luc, 1200 Brussels, Belgium

**Keywords:** fertility, premature ovarian insufficiency, diminished ovarian reserve, hormone replacement therapy, fertility preservation

## Abstract

Premature ovarian insufficiency (POI) and diminished ovarian reserve (DOR) are two related conditions characterized by a reduced ovarian reserve. Their etiologies are multifactorial, encompassing iatrogenic causes such as chemotherapy, pelvic surgery, or radiotherapy, as well as non-iatrogenic factors including genetic and chromosomal abnormalities, environmental exposures, autoimmune mechanisms and idiopathic sources. Early recognition of these conditions is essential, as timely and appropriate management can significantly impact both reproductive potential and long-term health. In women with POI, hormone replacement therapy is required to prevent the detrimental effects of estrogen deficiency on wellbeing and overall health, while in women with DOR, management focuses on counseling, fertility preservation when pregnancy is not an immediate goal, and strategies to optimize assisted reproductive outcomes when conception is desired. In addition, emerging research into ovarian rejuvenation offers promising new avenues for future therapeutic approaches. This review summarizes current knowledge on the pathophysiology, diagnosis, and management of POI and DOR, while highlighting innovative developments in reproductive medicine.

## 1. Introduction

The term ‘ovarian reserve’ refers biologically to the pool of primordial follicles (PMFs) established during fetal life, when primordial germ cells colonize the genital ridges and proliferate to generate approximately 7 million oocytes by midgestation, ~85% of which are lost before birth. Follicle numbers continue to decline throughout reproductive life, with only ~450 reaching ovulation and the rest undergoing atresia [1]. Serum anti-Müllerian hormone (AMH), while not produced by PMFs, serves as an indirect marker of their numbers and is widely used to estimate reproductive lifespan [2]. In clinical practice, the ovarian reserve is evaluated by AMH measurement, hormonal assays (follicle-stimulating hormone [FSH], estradiol) and antral follicle count (AFC) by ultrasound. It is important to note that AMH values are a poor predictive marker for natural pregnancies and should not be used to determine the chances of conception [3]. Decline in the ovarian reserve is a continuous process beginning during fetal life, with a progressive drop in the number of PMFs by menopause. Pathological alterations include premature ovarian insufficiency (POI) and a diminished ovarian reserve (DOR).

## 2. Premature Ovarian Insufficiency

POI is a subclass of ovarian dysfunction originating within the ovary. It is characterized by loss of ovarian function before the age of 40. The main symptom is amenorrhea or irregular menstrual cycles, along with elevated gonadotropins (FSH > 25 IU/L) and low estradiol levels [4]. The prevalence of POI before 40 years of age appears to be around 3.5% according to recent publications [5]. Not only do patients suffer from symptoms associated with estrogen deficiency like hot flushes, they also endure long-term consequences of low estrogen levels. These include loss of bone mineral density and impaired sexual function, cardiovascular and neurological health, all affecting their quality of life. Moreover, this disorder leads to infertility.

### 2.1. Diagnostic Criteria

**Figure 1:**
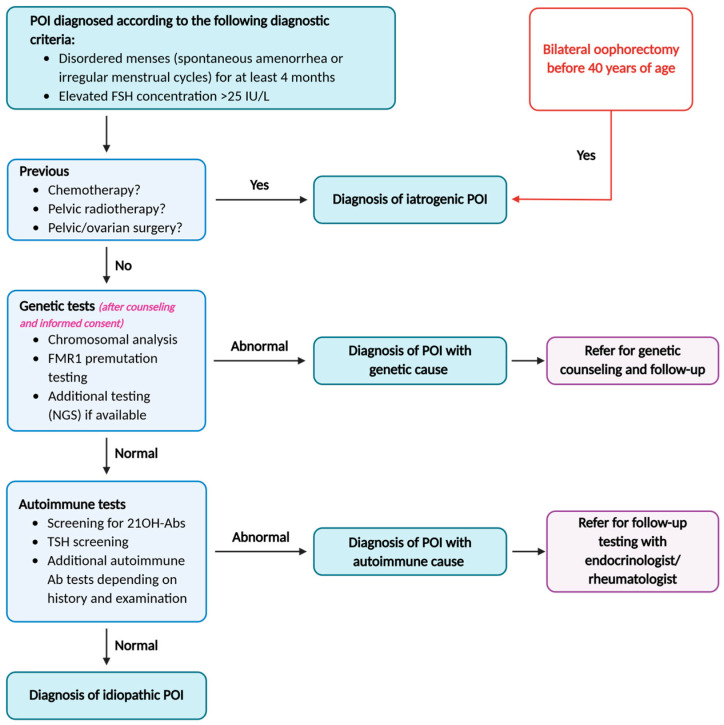
Summary of recommendations on POI diagnosis, as well as advised further testing to establish a cause for POI. 21OH-Abs = 21-hydroxylase autoantibodies, FMR1 = fragile X mental retardation 1, FSH = follicle-stimulating hormone, NGS = next-generation sequencing, TSH = thyroid stimulating hormone. Adapted from [4]. Created with BioRender.com. M. Dolmans (2025).

According to recently published international guidelines, POI diagnosis relies solely on the following criteria: disrupted menstrual cycles, namely spontaneous amenorrhea or irregular menstrual cycles for at least 4 months, with elevated FSH values of >25 IU/L. AMH evaluation is not recommended as a primary diagnostic test [4]. The summary of the recommendations on diagnosis of POI, as well as the recommended further testing to establish a cause for POI can be found in Figure 1.

### 2.2. Etiologies

#### 2.2.1. Iatrogenic Causes of POI

Chemotherapy is the primary and most common cause of POI, affecting the ovarian reserve by depletion of the PMF pool through a number of mechanisms. First, chemotherapy targets proliferating cells, making growing follicles the primary casualty of its effects. Although these follicles do not constitute the ovarian reserve per se, their destruction can cause transient amenorrhea during treatment, in other words short-term side effects [6]. PMFs, which constitute the actual ovarian reserve, are affected through accelerated and premature activation. This is mediated, on the one hand, by a reduction in AMH secreted by growing follicles, which normally acts to maintain PMF quiescence, and on the other, intrinsic activation of signaling pathways like PI3K-mTOR, driving follicle activation. Once activated, these follicles begin to grow and subsequently become vulnerable to any ongoing chemotherapy [7]. Finally, stromal damage has also been observed following chemotherapy administration [8,9]. Importantly, not all chemotherapy regimens negatively impact the ovarian reserve. The degree of gonadotoxicity depends on several factors: (i) the type of agent used, with alkylating agents being the most damaging; (ii) the cumulative dose and duration of treatment knowing that a cyclophosphamide-equivalent dose (CED) below 7500 mg/m^2^ is associated with a lower gonadotoxic risk; and (iii) the patient’s age, as prepubertal girls appear to be more resistant to chemotherapy-induced ovarian damage than postpubertal individuals [10]. Nevertheless, the extent of ovarian damage is highly variable from patient to patient. Some retain a normal ovarian reserve and fertility, while others may develop DOR, with or without compromised fertility or, in more severe cases, POI.

Pelvic radiotherapy has gonadal side effects in patients of all ages, but the degree of damage depends on the irradiation field, doses administered and schedule fractionation, the existing ovarian reserve and patient age. Oocytes are very sensitive to radiation and a dose of less than 2 Gy is sufficient to destroy 50% of PMFs [11], causing immediate or future POI. It is actually possible to determine the gonadotoxic dose of radiotherapy according to the age and ovarian reserve of the patient [12].

In young patients, pelvic and ovarian surgery for malignant gynecological diseases like endometrial, ovarian or cervical cancer that require bilateral salpingo-oophorectomy induce immediate menopause and subsequent irreversible infertility. Moreover, some benign gynecological tumors like endometriomas also require surgery, which can be harmful to the ovarian reserve if repeated multiple times or not performed properly, as endometrioma surgery is technically difficult. Indeed, theses cysts do not have a clear dissection plane [13], resulting in ablation of healthy ovarian tissue containing follicles in more than 60% of cases [14,15]. According to the literature, surgery for endometriomas is the most deleterious ovarian surgery for cysts [16].

#### 2.2.2. Non-Iatrogenic Causes of POI

**Figure 2:**
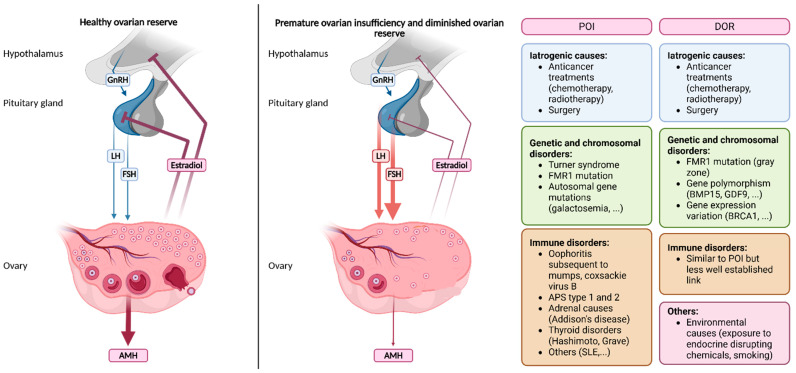
(**Left**): Healthy ovarian reserve, growing follicles secrete anti-Müllerian hormone (AMH) and estradiol, which exerts negative feedback on the hypothalamus and pituitary gland, generating low levels of follicle-stimulating hormone (FSH) and luteinizing hormone (LH). (**Right**): Decreased ovarian reserve shows lower AMH, lower estradiol and higher FSH and LH levels. Created with Biorender (M. Dolmans, 2025).

When non-iatrogenic, POI generally arises from either depletion or dysfunction of ovarian follicles for different reasons (Figure 2).

##### Genetic and Chromosomal Disorders

Women with structural and/or numerical anomalies of the X chromosome make up the largest subgroup of individuals with non-iatrogenic POI. Turner syndrome patients (45,X) account for 4–5% of all POI subjects. Depletion of their ovarian reserve is caused by loss of the X chromosome as, unlike most somatic cells, oocytes need two active X chromosomes to differentiate. This results in spontaneous accelerated follicle loss and oocyte depletion within the first 10 years of life [17]. In case of mosaic Turner syndrome (45,X/46,XX), menarche can occur followed by menstruation for several years [18,19].

After Turner syndrome, fragile X mental retardation 1 (FMR1) is the most common congenital cause of POI (2–5% of women with POI). Expansion to more than 200 repeats of the CGC trinucleotide at the 5′ end of FMR1 leads to fragile X syndrome. However, patients with the premutation, namely fewer repeats of the trinucleotide, can also present with POI. Indeed, the risk of POI correlates with the number of repeats, and higher numbers are associated with an increased likelihood of POI [20]. Mechanistically, FMR1 premutation leads to toxic gain-of-function effects from expanded CGG-repeat mRNA, which sequesters RNA-binding proteins and disrupts granulosa cell function, contributing to follicle depletion and an impaired ovarian response [21]. Dysregulation of AMH expression in granulosa cells has also been observed, suggesting a compensatory response to follicle loss [22].

There are countless autosomal gene mutations that are potentially involved in inducing POI. Galactosemia, a genetic disorder provoked by a deficiency in galactokinase that causes accumulation of toxic galactose or in galactose-1-phosphate uridyltransferase that leads to aggregation of toxic galactose-1-phosphate, is known to trigger POI. The suspected mechanism here is increased oocyte and stromal cell apoptosis. In case of many other mutations, however, the exact source in the physiopathology of POI is unknown [23].

##### Immune Disorders

The blood-follicle barrier, where granulosa cells grow in an avascular environment and provide oocytes with nutrients, confers a certain degree of protection against immune reactions to the germ line. Nevertheless, in some cases, this barrier is no longer effective, like in viral infections with ovarian tropism or autoimmune responses resulting in immune-mediated ovarian damage. This immune reaction is known as oophoritis and is characterized by different levels of infiltration of the innate and adaptive immune system in the theca layer of growing follicles [24].

Some viral infections like mumps [25] and coxsackie virus B [26] can trigger ovarian oophoritis. Autoimmune diseases can also provoke immune processes against ovarian tissue, characterized by the presence of oophoritis and/or anti-ovarian antibodies. Indeed, about 30% of POI patients have been previously diagnosed with other concomitant autoimmune diseases.

The most common cause of autoimmune POI is autoimmune polyglandular syndrome type 1 and type 2, activated by mutations in the AIRE gene regulating immunological tolerance and showing autoimmunity against two or more endocrine organs [27]. Adrenal autoimmunity like Addison’s disease is highly prevalent in patients with POI due to cross-reactivity of autoantibodies like 21-hydroxylase that act on steroid-producing cells [24,28]. Finally, POI can also be associated with disorders of the thyroid (like hypothyroidism, Hashimoto’s thyroiditis and Grave’s disease) and other autoimmune conditions like diabetes mellitus, rheumatoid arthritis and systemic lupus erythematosus [29]. Although thyroid peroxidase (TPO) antibody screening is often performed, it is not recommended because of the high prevalence of positive TPO antibodies in the general population. The 21-hydroxylase autoantibody is the only one endorsed for screening in case of suspected autoimmune POI [4].

## 3. Diminished Ovarian Reserve

Diminution of the ovarian reserve is influenced by age, genetics and environmental variables. While it is challenging to predict the rate of an individual’s ovarian reserve decline, clinicians are often asked for advice about fertility potential and/or recommendations on fertility treatment options [30].

DOR can be physiological, when it is age-related and happens in a woman’s mid-40s, or pathologic when onset occurs at a younger age. However, there is no strict consensus on the age limit, as we know that POI is defined by amenorrhea before the age of 40, but both DOR and POI can arise within a spectrum. DOR may be considered an early sign of advanced ovarian aging in young women, preceding the onset of cycle irregularity prior to establishment of real POI. While approximately 10% of women below the age of 40 years may experience DOR, most of them will not experience POI [31,32]. Young patients with DOR often face challenges when trying to conceive, especially if assisted reproductive technology (ART) is needed for other reasons like male infertility.

### 3.1. Diagnostic Criteria

DOR is defined based on a diminished AFC (<5), lower serum AMH levels (<0.5–1 ng/mL) and/or elevated FSH values (>10 IU/L on cycle days 2–4), creating a hypergonadotropic hormonal environment that does not correspond to the strict definition of POI [31,32,33].

### 3.2. Etiologies

DOR can have multiple etiologies, including genetic, autoimmune, idiopathic and iatrogenic. Of course, the principal cause is the decline in the ovarian reserve associated with age. Indeed, at some point in every woman’s reproductive life, she will meet the criteria for DOR upon approaching menopause. Age-related DOR is therefore considered physiological [34].

#### 3.2.1. Iatrogenic Causes of DOR

As explained above for POI, chemotherapy, radiotherapy, and ovarian surgery are common causes of DOR, and their effect on the ovary can be a continuum. In some cases, these treatments lead directly to POI, while in others they induce ‘only’ DOR. The incidence of POI in childhood cancer survivors is just 8–10%, but those who do retain ovarian function after treatment may nevertheless exhibit decreased fertility rates [35].

#### 3.2.2. Non-Iatrogenic Causes of DOR

Reasons for prematurely lower numbers of follicles can manifest at many different stages of follicle and oocyte development. It can simply be a matter of a small pool of PMFs due to reduced production of oocytes embryologically, or abnormal follicle development and increased atresia [31].

##### Genetic and Chromosomal Disorders

Multiple genes play roles in folliculogenesis. One of the known genetic causes of POI, the FMR1 mutation, is also thought to play a role in DOR. Indeed, compared to healthy subjects, DOR patients stand a greater chance of being diagnosed as premutation carriers or being in the ‘gray zone’, meaning that they carry between 35 and 54 CGG triplet repeats [36,37]. Many gene polymorphisms are associated with DOR, such as BMP15 and GDF9, both related to follicle development [38]. The inheritance pattern for both is autosomal recessive for loss-of-function variants (frequency of <1% for BMP15 and 2.8% in the POI/DOR population for GDF9), while most heterozygous missense variants are considered benign or of uncertain significance [39,40]. The incidence of BRCA1 mutations in the general population is approximately 1 in 400 to 1 in 800 women [41,42].

On the other hand, variations in expression of other genes, like BRCA1 or 2 mutations, can also trigger DOR [43,44]. Among women with DOR or POI, the prevalence of BRCA1 mutations is higher than in the general population, but remains relatively low. Meta-analyses and cohort studies indicate that BRCA1 mutations are present in up to 4–6% of women with DOR and the association between BRCA1 mutations and DOR is supported by consistently lower AMH levels and reduced oocyte yield in carriers [45,46]. These variations contribute to DOR mainly through impaired DNA double-strand break repair in oocytes, leading to accelerated loss of PMFs and increased oocyte apoptosis. BRCA1 is essential for homologous recombination repair and pathogenic variants result in accumulation of DNA damage within oocytes, which accelerates follicle depletion and ovarian aging [41,47].

##### Immune Disorders

Autoimmune causes of POI have been widely documented, but its link to DOR is less well established, even if the two conditions can happen in a continuum. A number of studies have suggested an autoimmune association with DOR, as in case of systemic lupus erythematosus where, despite mild disease activity, patients exhibit a significantly smaller ovarian reserve (assessed by AMH values) than age-matched healthy women [48,49,50]. However, in the study by Lawrenz et al., there was no significant difference in terms of pregnancy or number of offspring between systemic lupus erythematosus-affected and healthy patients [48]. As in case of POI, autoimmune polyglandular syndrome type 1 has been associated with the presence of DOR, which could precede the onset of POI [51].

##### Environmental Causes of DOR

Epigenetics defines molecular modifications in DNA implicated in gene expression and function, which are inheritable but do not alter the DNA sequence [52,53]. The most well-known of these are DNA methylation, histones and post-translational modifications. These changes can be caused by the environment and endocrine-disrupting chemicals (EDCs) like vinclozolin (fungicide), methoxychlor (pesticide), genistein (phytoestrogen), bisphenol A (plasticizer), parabens and per- and polyfluoroalkyl substances (PFAS). Animal studies previously revealed that while EDCs affected the fertility of exposed female mice, they also affected future generations, causing differential methylation patterns and pointing to epigenetic effects. These studies suggest that environmental toxins influencing the epigenome may create similar DOR phenotypes in humans [54,55]. Many environmental compounds can bind to the estrogen receptor (albeit at lower affinities) thanks to the similarity with natural ligands. They therefore have the potential to impact either the initial setting of the ovarian reserve during development or its trajectory during adult life [56]. However, investigations into single EDC effects on the ovary have often used higher doses than those encountered in real life [57], where people are often exposed to a mixture of EDCs at relatively low levels. Studies using EDC concentrations in follicular fluid as exposure markers showed that aggregates of EDCs significantly increased the likelihood of DOR compared to each EDC’s individual effect. Bisphenol A has long been known to have negative repercussions on ovarian function among other deleterious ramifications, so its use has declined. It was recently found that other phenols like BP4 are also linked to DOR, but more research is needed to confirm these results [58]. Higher urinary concentrations of some phthalates used in the production of plastics and cosmetics were associated with DOR as evidenced by significantly decreased AFCs [59]. While PFAS affect reproductive health in disorders like polycystic ovary syndrome or uterine myomas [60], their impact on the ovarian reserve is still unclear [61]. With regard to parabens, although a few animal studies have reported toxicity to the reproductive system, their effect on human ovarian function remains to be elucidated [58].

Finally, smoking is a well-established cause of DOR [62]. Indeed, studies have shown that smoking may induce direct oocyte toxicity, leading to accelerated follicle depletion and subsequent DOR. Women who smoke cigarettes are more likely to experience irregular menses, have poorer corpus luteum function and enter menopause up to 4 years sooner than their nonsmoking counterparts.

## 4. Therapeutic Options and Management

### 4.1. Fertility Preservation Strategies When POI or DOR Are Anticipated

When POI and DOR are anticipated due to planned treatments (including chemotherapy and/or radiotherapy and surgery) or because of known conditions like Turner syndrome, fertility preservation has to be offered before the onset of POI/DOR.

Indeed, there are a number of indications for fertility preservation, including oncological, non-oncological and elective. The most common oncological indications are hematological malignancies (Hodgkin’s lymphoma, non-Hodgkin’s lymphoma and leukemia) and breast cancer because of their high incidence among the younger population [63]. Other indications include sarcoma, colorectal carcinoma and central nervous system tumors, which all require chemotherapy and/or radiotherapy, significantly increasing the risk of long-term fertility impairment [10,64]. Fertility preservation should also be offered to women affected by non-oncological conditions that call for chemotherapy and/or radiotherapy, like certain hematological diseases (thalassemia, sickle cell disease, aplastic anemia) requiring bone marrow transplantation, or autoimmune disorders (systemic lupus erythematosus, rheumatoid arthritis) needing low-dose treatments with alkylating agents [10]. Other benign diseases can impair fertility when therapy involves multiple ovarian surgeries, increasing the risk of iatrogenic damage. These include borderline ovarian tumors, recurrent or bilateral benign ovarian tumors, recurrent ovarian torsion and ovarian endometriosis, especially bilateral endometriomas or recurrent endometriomas after surgery, fast-growing endometriotic lesions or disease occurrence at a very young age [65]. Genetic conditions causing POI, such as Turner syndrome, can also be indications for fertility preservation [17].

Just as the reasons for fertility preservation differ, so do the approaches. Currently, they include oocyte and embryo cryopreservation, as well as ovarian tissue cryopreservation followed by ovarian tissue transplantation [10].

### 4.2. POI Management: Hormone Replacement Therapy

When patients present with complete POI, there are limited to no therapeutic options to preserve their remaining ovarian reserve and achieve spontaneous pregnancy. Some controversial treatments have been proposed, like corticosteroids in case of autoimmune etiology [66,67], but there is no proof of their efficacy.

POI has multisystemic effects with physical as well as emotional impacts. It is therefore of utmost importance to encourage patients to pursue a healthy lifestyle, including a well-balanced diet, with physical activity to maintain a healthy weight range [68,69]. Alongside lifestyle management, the most important treatment for women with POI is appropriate hormone replacement therapy (HRT), which should be initiated as soon as the diagnosis is made and continued at least until the normal age of menopause (51.5 years) at higher doses than typical postmenopausal HRT [4]. Indeed, HRT is useful not only to alleviate symptoms of estrogen deficiency, including vasomotor symptoms like hot flushes and sweats, and urogenital problems like vaginal dryness and recurrent urinary tract infections, but also exert beneficial effects on the overall quality of life by improving mood/cognitive issues, energy levels, and musculoskeletal aches and pain [68]. Most importantly, HRT minimizes long-term health consequences like osteoporosis and cardiovascular diseases. Moreover, in young girls, HRT appears to aid the development of secondary sexual characteristics, like uterine growth, despite a lack of clarity in the current literature regarding the latter [70].

As described by Panay et al., to achieve the best outcomes with POI while keeping side effects/risks to a minimum, three principles should be respected for HRT administration: (i) replaced hormones should be identical to those lacking; (ii) non-oral estrogen delivery routes are better at avoiding first-pass hepatic metabolism, hence lessening the prothrombotic effect of oral estrogen; and (iii) estrogen doses should be generally higher than those used in natural menopause [68]. This makes 75–100 µg estradiol patches or 3–4 doses of 0.75 mg of estrogen gel (as opposed to 1–2 doses in normal menopause management) the ideal therapeutic option. The transdermal approach is also easier to monitor and adapt in case of side effects like mastalgia or migraine when the patient cannot tolerate higher doses. Of course, the endometrium has to be protected if the patient still has a uterus by using progesterone (200 mg micronized progesterone orally or vaginally for 12 days per cycle). This combination is not prothrombotic and is associated with the lowest breast cancer risk compared to other HRT combinations [71]. Other combinations may be effective too, but all therapies should be discussed with patients, and treatments personalized according to their symptoms and expectations.

### 4.3. DOR Management: Improving Ovarian Stimulation Protocols

Despite having a low ovarian reserve, DOR patients do not require HRT, as their ovaries still produce estradiol. If they wish to conceive, these women should be advised not to delay pregnancy. Although DOR patients do not necessarily present with infertility, if they need ART, they frequently face challenges associated with ovarian stimulation. As their ovarian reserve is small, typical stimulation protocols for IVF often result in a poor oocyte yield. There are two main reasons why numbers of collected oocytes may be limited. The first is linked to the internal capacity of the patient to actually provide oocytes, having a diminished ovarian stockpile. The second is a hyporesponse, where ovarian capacity is higher, but stimulation is not optimal, generating a poor ovarian response [72]. Different therapeutic strategies have been proposed to manage DOR patients, but with mixed results. Overall, according to a recent systematic review with a meta-analysis, some interventions might enhance ovarian output and pregnancy rates in women with DOR [73].

#### 4.3.1. Dehydroepiandrosterone (DHEA) and Testosterone

Androgens increase granulosa cell FSH receptor expression, boosting FSH effects during ovarian stimulation, thereby improving the ovarian response [74,75,76,77]. However, while use of DHEA was found to increase the number of retrieved mature oocytes, it did not raise live birth rates [73,78,79,80,81]. Testosterone supplementation, on the other hand, did boost live birth rates in DOR patients [82,83,84], but the mechanism involved remains unclear. Its effect on endometrial receptivity is still being debated, and better pregnancy rates in women treated with testosterone are probably related to the impact on recruited ovarian follicles than embryo quality and/or implantation [73].

#### 4.3.2. High-Dose Gonadotropins vs. Mild Stimulation

Use of high-dose gonadotropins as opposed to mild stimulation in DOR patients remains a contentious issue. Indeed, it seems intuitive to increase stimulation in women with a small ovarian reserve, so three randomized controlled trials compared low-dose vs. high-dose regimens in women with DOR [85,86,87]. None demonstrated any positive impact of higher FSH doses on live birth rates. The first trial showed that DOR patients do not benefit from greater gonadotropin stimulation in terms of mature oocyte yield or clinical pregnancy rates [85]. The other two trials did establish an increase in the number of oocytes retrieved through high-dose stimulation compared to low-dose stimulation, but similar pregnancy rates [86,87]. Overall, a meta-analysis pooling the results of all three randomized controlled trials ascertained that fewer oocytes are retrieved from DOR patients undergoing mild stimulation than from those subjected to standard high-dose ovarian stimulation protocols. Nonetheless, clinical pregnancy, live birth and miscarriage rates were similar between groups [73]. 

There are many other therapeutic strategies seeking to improve ovarian stimulation outcomes, like luteinizing hormone co-treatment prior to ovarian stimulation [88,89], dual-stimulation protocols [90] or addition of growth hormone [91,92]. So far, none have proved effective, and more robust evidence is needed before they can be implemented.

### 4.4. Potential Therapeutic Options to Boost the Ovarian Reserve

#### 4.4.1. In Vitro Activation

It is estimated that three out of four women with POI have dormant PMFs remaining in their ovaries [93], explaining why POI sufferers still have a 5% chance of spontaneous pregnancy [94]. Recently, in vitro activation of remaining follicles was proposed to boost the chances of pregnancy in recent-onset POI and DOR patients. This procedure involves laparoscopic removal of ovarian cortical tissue and its fragmentation to disrupt the Hippo signaling pathway, before incubation with pharmacological agents (like PI3K/Akt stimulators) to further activate dormant follicles. The tissue is then autotransplanted and ovarian stimulation initiated to promote follicle growth and oocyte retrieval for IVF. This dual approach targets both mechanical and biochemical pathways to maximize follicle activation [95,96].

Drug-free in vitro activation is a simplified procedure, with no pharmacological incubation, relying solely on mechanical fragmentation of ovarian cortex to activate follicles through the Hippo pathway. Using drug-free in vitro activation, Kawamura et al. were able to demonstrate increased antral follicle numbers and more oocytes retrieved when patients were stimulated for IVF [97,98]. However, this study remains experimental and since publication of these results, other studies replicating the method have yielded negative outcomes [99] and others positive [100,101,102]. Concerns have also emerged, as the technique is quite invasive and affects the ovary itself [103,104].

#### 4.4.2. Platelet-Rich Plasma Injections

There are many new experimental approaches looking to rejuvenate the ovary. Indeed, in a recent study, women with DOR who had previously had a poor response to IVF treatments showed better ovarian reserve parameters when treated with intraovarian injections of autologous platelet-rich plasma [105]. One nonrandomized interventional study was conducted with a control group and exhibited higher AMH, AFC and live birth rates [106], while another found no improvement in these parameters after injection in patients presenting with a poor ovarian response, findings which may not be generalizable to POI patients [107]. Yet another recent retrospective study also showed no beneficial effect of platelet-rich plasma for POI patients [108]. Two recent systematic reviews with meta-analyses of 38 and 23 prospective observational studies, respectively, concluded that platelet-rich plasma treatment could enhance ovarian reserve testing parameters (higher AFC, AMH, oocyte count and embryo count, and lower FSH levels) in a comparison of values before and after treatment [109,110]. Nevertheless, more trials are essential before this approach can be used in a clinical context.

#### 4.4.3. Bone Marrow Stem Cell Transplantation

The third promising technique is bone marrow stem cell transplantation to ovarian tissue through catheterization. Stem cells secrete growth factors involved in the cell cycle, gene expression and follicle activation pathways (Hippo and PI3K/Akt). In one study, this technique resulted in enhanced ovarian function in 81% of poor responders (out of 17 patients in total), increased AMH and AFC, and retrieval of more follicles and oocytes, leading to 3 spontaneous pregnancies and 3 IVF pregnancies [111]. Other studies have been conducted [112], but randomized controlled trials with greater numbers of patients are needed. Recent publications have also shown the effectiveness of stem cell-derived extracellular vesicles, but these studies are only on animal models [113] and require further investigations in human setting.

#### 4.4.4. Hope from Experimental Studies

Preclinical experimental studies using mainly murine models have shown potentially beneficial effects of mesenchymal stem cell (MSC) therapy. Indeed, in the context of ovarian recovery and gonadotoxic damage healing, like after administration of gonadotoxic chemotherapy, MSC-based treatments have yielded consistently encouraging results. Increased follicle counts and improved ovarian function have been achieved primarily through modulation of the ovarian microenvironment and reduction in apoptosis in granulosa cells, theca cells and stromal cells [114,115,116]. These effects are likely mediated by MSC-derived growth factors, such as vascular endothelial growth factor, hepatocyte growth factor and insulin-like growth factor-1, which are known to support cell survival, angiogenesis and proliferation [117,118]. MSC-derived exosomes have also proved effective at restoring ovarian function in case of chemotherapy-induced POI, so this regenerating effect does not only originate from growth factors, but also other components secreted by MSCs. Indeed, in animal models of POI, exosome treatment has boosted follicle populations, corpus luteum formation and numbers of offspring compared to untreated animals [113,119,120,121,122]. These studies give hope as new methods emerge to rejuvenate the ovarian reserve.

## 5. Conclusions

POI and DOR both reside along a continuum of a dwindling ovarian reserve, making early diagnosis essential. While iatrogenic causes are most common, identifying the underlying etiology in non-iatrogenic cases remains critical to guide additional treatment in autoimmune conditions and provide appropriate genetic counseling in chromosomal abnormalities. In POI, timely detection enables early initiation of HRT, which is crucial to prevent long-term health consequences and should be maintained until the average age of natural menopause. Clearly, accurate diagnosis before starting HRT is crucial to avoid potential deleterious effects on women’s psychological and physical health. When POI is anticipated, as in the context of chemotherapy or surgery, fertility preservation strategies must be offered. In DOR, early recognition allows for individualized counseling and timely consideration of fertility preservation options or reproductive planning. Ultimately, early detection and tailored management are key to optimizing both long-term health and reproductive outcomes.

## Data Availability

Not applicable.
